# Supplementary material for: Substantial loss of trawlable biomass and lack of recovery in a marine ecosystem
Source: Commun Biol. 2025 May 30;8:831. doi: 10.1038/s42003-025-08240-3 (PMC12125391; doi:10.1038/s42003-025-08240-3)
Supplement: Supplementary file 2 — Supplementary Information [file 42003_2025_8240_MOESM2_ESM.pdf]

Supplementary Material

Supplementary Table 1. List of all species used in this study. Also includes the zonal strata (P: Pelagic, D: Demersal) and if the species is under a commercial fishery as of April 2020.

Latin name	English name	Zonal strata	Com. species
Alosa pseudoharengus	Alewife	P	x
Alosa sapidissima	American shad	P	
Amblyraja radiata	Thorny skate	D	x
Ammodytes dubius	Northern sand lance	D	
Anarhichas denticulatus	Northern wolffish	D	
Anarhichas lupus	Striped Atlantic wolffish	D	
Anarhichas minor	Spotted wolffish	D	
Anguilla rostrata	American eel	D	
Apeltes quadracus	Four-spine stickleback	D	
Argentina silus	Greater argentine	D	
Artediellus atlanticus	Atlantic hookear sculpin	D	
Artediellus uncinatus	Arctic hookear sculpin	D	
Aspidophoroides monopterygius	Alligatorfish	D	
Aspidophoroides olriki	Arctic alligatorfish	D	
Bathyraja spinicauda	Spinytail skate	D	
Boreogadus saida	Arctic cod	P	
Brevoortia tyrannus	Atlantic menhaden	P	
Caelorinchus caelorinchus	Longnose grenadier	D	
Cancer borealis	Jonah crab	D	
Cancer irroratus	Atlantic rock crab	D	
Careproctus longipinnis	Longfin seasnail	D	
Careproctus reinhardti	Sea tadpole	D	
Centroscyllium fabricii	Black dogfish	D	
Chaceon quinquedens	Red deepsea crab	D	
Chauliodus sloani	Sloane's viperfish	D	
Chionoecetes opilio	Snow crab	D	x
Clupea harengus	Atlantic herring	P	x
Coryphaenoides rupestris	Roundnose grenadier	D	
Cottunculus microps	Polar sculpin	D	
Cottunculus thompsoni	Pallid sculpin	D	
Cryptacanthodes maculatus	Wrymouth	D	
Cyclopterus lumpus	Lumpfish	D	
Cyclothone microdon	Veiled anglemouth	D	
Decapoda spp.	Shrimps	na	x
Dipturus laevis	Barndoor skate	D	
Enchelyopus cimbrius	Fourbeard rockling	D	
Eumesogrammus praecisus	Fourline snakeblenny	D	
Eumicrotremus derjugini	Leatherfin lumpsucker	D	
Eumicrotremus spinosus	Atlantic spiny lumpsucker	D	
Gadus morhua	Atlantic cod	D	x
Gadus ogac	Greenland cod	D	
Gaidropsarus ensis	Threebeard rockling	D	
Gasterosteus aculeatus	Three-spine stickleback	D	
Glyptocephalus cynoglossus	Witch flounder	D	x
Gymnelis viridis	Fish doctor	D	
Gymnocanthus tricuspis	Arctic staghorn sculpin	D	
Helicolenus dactylopterus	Blackbelly rosefish	D	
Hemitripterus americanus	Sea raven	D	
Hippoglossoides platessoides	American plaice	D	x
Hippoglossus hippoglossus	Atlantic halibut	D	x
Homarus americanus	American lobster	D	x
Hyas araneus	Toad crab	D	
Icelus bicornis	Twohorn sculpin	D	
Icelus spatula	Spatulate sculpin	D	
Lampadena speculigera	Mirror lanternfish	P	
Leptagonus decagonus	Atlantic sea poacher	D	
Leucoraja erinacea	Little skate	D	
Leucoraja ocellata	Winter skate	D	x
Limanda ferruginea	Yellowtail flounder	D	x
Liparis atlanticus	Atlantic seasnail	D	
Liparis coheni	Gulf snailfish	D	
Liparis fabricii	Gelatinous snailfish	D	
Liparis gibbus	Variegated snailfish	D	
Liparis liparis	Striped seasnail	D	
Liparis tunicatus	Greenland seasnail	D	
Lithodes maja	Northern stone crab	D	
Lophius americanus	American angler	D	
Lumpenus fabricii	Slender eelblenny	D	
Lumpenus lumpretaeformis	Snake blenny	D	
Lumpenus maculatus	Daubed shanny	D	
Lumpenus medius	Stout eelblenny	D	
Lycenchelys paxillus	Common wolf eel	D	
Lycenchelys verrilli	Wolf eelpout	D	
Lycodes esmarki	Esmark's eelpout	D	
Lycodes lavalaei	Laval's eelpout	D	
Lycodes pallidus	Pale eelpout	D	
Lycodes polaris	Polar eelpout	D	
Lycodes reticulatus	Arctic eelpout	D	
Lycodes terraenova	Atlantic eelpout	D	
Lycodes vahlii	Vahl's eelpout	D	
Macrozoarces americanus	Ocean pout	D	
Malacoraja senta	Smooth skate	D	x
Mallotus villosus	Capelin	P	x
Melanogrammus aeglefinus	Haddock	D	x

Latin name	English name	Zonal strata	Com. species
Melanostigma atlanticum	Atlantic soft pout	D	
Menidia menidia	Atlantic silverside	P	x
Merluccius bilinearis	Silver hake	D	
Microgadus tomcod	Atlantic tomcod	D	
Morone saxatilis	Striped bass	D	
Myoxocephalus aenaeus	Grubby	D	
Myoxocephalus octodecemspinosus	Longhorn sculpin	D	
Myoxocephalus scorpioides	Arctic sculpin	D	
Neolithodes grimaldii	Porcupine crab	D	
Nezumia bairdii	Marlin-spike grenadier	D	
Notolepis rissoi	White barracudina	P	
Osmerus mordax	Rainbow smelt	P	x
Paralepis coregonoides	Paralepis coregonoides	P	
Paraliparis calidus	Lowfin snailfish	D	
Peprilus triacanthus	American butterfish	P	
Petromyzon marinus	Sea lamprey	D	
Pholis fasciata	Banded gunnel	D	
Pholis gunnellus	Rock gunnel	D	
Pollachius virens	Pollock	D	x
Pseudophichthus splendens	Pseudophichthus splendens	D	
Pseudopleuronectes americanus	Winter flounder	D	x
Reinhardtius hippoglossoides	Greenland halibut	D	x
Rouleina maderensis	Madeiran smooth-head	D	
Salmo salar	Atlantic salmon	P	
Scomber scombrus	Atlantic mackerel	P	x
Scomberesox saurus	Atlantic saury	P	
Scophthalmus aquosus	Windowpane flounder	D	
Sebastes mentella	Deepwater Redfish	D	
Sebastes sp.	Redfish unidentified	D	
Squalus acanthias	Spiny dogfish	D	
Stichaeus punctatus	Arctic shanny	D	
Stomias boa	Boa dragonfish	D	
Tautogolabrus adspersus	Cunner	D	
Triglops murrayi	Mailed sculpin	D	
Ulvaria subbifurcata	Radiated shanny	D	
Urophycis chesteri	Longfin hake	D	
Urophycis chuss	Red hake	D	
Urophycis tenuis	White hake	D	x

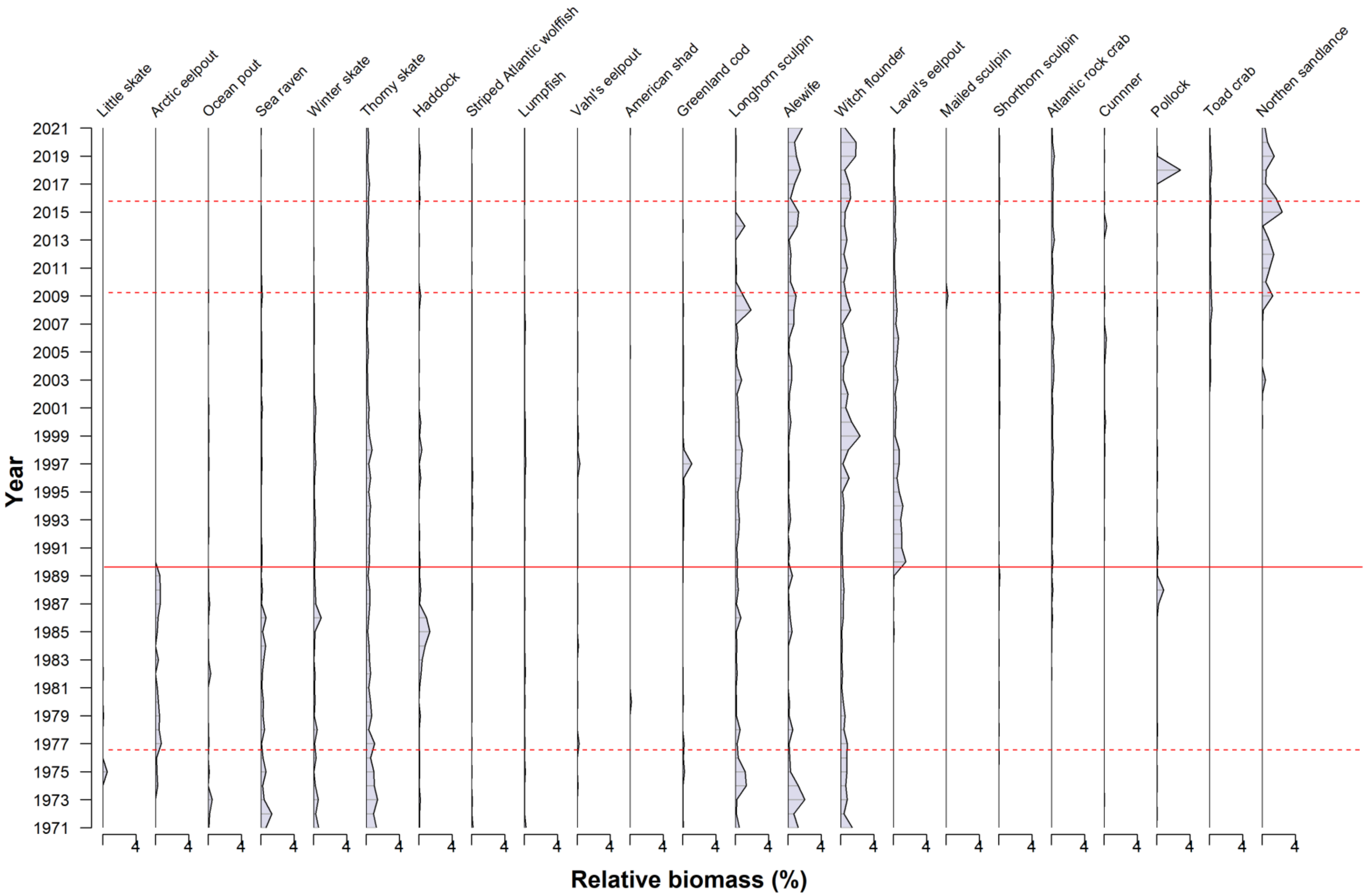

Supplementary Figure 1. The relative biomass of 23 taxa in addition to the 17 taxa with highest relative abundances shown in Figure 3. These taxa have a relative biomass of between 0.17 – 3.39 % biomass in at least one year across the time series. The solid red line indicates the regime shift identified in the study and the dotted lines denote each identified subperiod.
