# Supplementary material for: Substantial loss of trawlable biomass and lack of recovery in a marine ecosystem
Source: Commun Biol. 2025 May 30;8:831. doi: 10.1038/s42003-025-08240-3 (PMC12125391; doi:10.1038/s42003-025-08240-3)
Supplement: Supplementary file 3 — Description of Additional Supplementary Files [file 42003_2025_8240_MOESM3_ESM.pdf]

# Description of Additional Supplementary Files

File name: Supplementary Data 1.

Description: Source data for Figure 2.

File name: Supplementary Data 2.

Description: Source data for Figure 3.

File name: Supplementary Data 3.

Description: Source data for Figure 4
