# Supplementary material for: Substantial loss of trawlable biomass and lack of recovery in a marine ecosystem
Source: Commun Biol. 2025 May 30;8:831. doi: 10.1038/s42003-025-08240-3 (PMC12125391; doi:10.1038/s42003-025-08240-3)
Supplement: Supplementary file 7 — Reporting Summary [file 42003_2025_8240_MOESM7_ESM.pdf]

nature portfolio

Corresponding author(s): Jacob Burbank

Last updated by author(s): May 2, 2025

Reporting Summary

Nature Portfolio wishes to improve the reproducibility of the work that we publish. This form provides structure for consistency and transparency in reporting. For further information on Nature Portfolio policies, see our Editorial Policies and the Editorial Policy Checklist.

Statistics

For all statistical analyses, confirm that the following items are present in the figure legend, table legend, main text, or Methods section.

n/a	Confirmed	
☐	☒	The exact sample size (*n*) for each experimental group/condition, given as a discrete number and unit of measurement
☒	☐	A statement on whether measurements were taken from distinct samples or whether the same sample was measured repeatedly
☐	☒	The statistical test(s) used AND whether they are one- or two-sided<br>*Only common tests should be described solely by name; describe more complex techniques in the Methods section.*
☐	☒	A description of all covariates tested
☐	☒	A description of any assumptions or corrections, such as tests of normality and adjustment for multiple comparisons
☐	☒	A full description of the statistical parameters including central tendency (e.g. means) or other basic estimates (e.g. regression coefficient) AND variation (e.g. standard deviation) or associated estimates of uncertainty (e.g. confidence intervals)
☒	☐	For null hypothesis testing, the test statistic (e.g. *F*, *t*, *r*) with confidence intervals, effect sizes, degrees of freedom and *P* value noted<br>*Give P values as exact values whenever suitable.*
☒	☐	For Bayesian analysis, information on the choice of priors and Markov chain Monte Carlo settings
☐	☒	For hierarchical and complex designs, identification of the appropriate level for tests and full reporting of outcomes
☒	☐	Estimates of effect sizes (e.g. Cohen's *d*, Pearson's *r*), indicating how they were calculated

Our web collection on statistics for biologists contains articles on many of the points above.

Software and code

Policy information about availability of computer code

Data collection	No software was used to collect data.
Data analysis	All analyses were conducted in the software environments C2 and R version 4.2.2, and the package "rioja" was used for cluster analyses. The data used in this paper are available on the Government of Canada Open Data Portal.

For manuscripts utilizing custom algorithms or software that are central to the research but not yet described in published literature, software must be made available to editors and reviewers. We strongly encourage code deposition in a community repository (e.g. GitHub). See the Nature Portfolio guidelines for submitting code & software for further information.

Data

Policy information about availability of data

All manuscripts must include a data availability statement. This statement should provide the following information, where applicable:

- Accession codes, unique identifiers, or web links for publicly available datasets
- A description of any restrictions on data availability
- For clinical datasets or third party data, please ensure that the statement adheres to our policy

The data used in this paper are available on the Government of Canada Open Data Portal (under the project name "Southern Gulf of St. Lawrence Ecosystem Research Vessel Survey (September survey, NAFO Division 4T) Dataset" (https://open.canada.ca/data/en/dataset/1989de32-bc5d-c696-879c-54d422438e64) . All code used for this study is available on the corresponding authors GitHub (https://github.com/jakeburb/SustainedLossofBiomass).

Research involving human participants, their data, or biological material

Policy information about studies with human participants or human data. See also policy information about sex, gender (identity/presentation), and sexual orientation and race, ethnicity and racism.

Reporting on sex and gender	NA
Reporting on race, ethnicity, or other socially relevant groupings	NA
Population characteristics	NA
Recruitment	NA
Ethics oversight	NA

Note that full information on the approval of the study protocol must also be provided in the manuscript.

Field-specific reporting

Please select the one below that is the best fit for your research. If you are not sure, read the appropriate sections before making your selection.

☐ Life sciences ☐ Behavioural & social sciences ☒ Ecological, evolutionary & environmental sciences

For a reference copy of the document with all sections, see nature.com/documents/nr-reporting-summary-flat.pdf

Ecological, evolutionary & environmental sciences study design

All studies must disclose on these points even when the disclosure is negative.

Study description	This study focuses on the sGSL, which is a shallow semi-enclosed sea that covers an area of approximately 85,000 km² south of the deep Laurentian Channel and is bordered by four Canadian provinces, Quebec, New Brunswick, Nova Scotia and Prince Edward Island (Figure 1). The abundance and biomass of the taxa, and the community structure of the marine ecosystem throughout the sGSL was determined using the data collected during the September multi-species bottom-trawl survey conducted by the DFO since 1971.
Research sample	This survey follows a stratified random sampling design, which covers an area of 73,214 km² and includes sampling of fish and invertebrates using a bottom trawl (Figure 1). The trawl survey was designed to provide biomass and abundance trends for fish and invertebrates distributed between depths of about 20 m to 350 m.
Sampling strategy	This survey follows a stratified random sampling design, which covers an area of 73,214 km² and includes sampling of fish and invertebrates using a bottom trawl (Figure 1). The trawl survey was designed to provide biomass and abundance trends for fish and invertebrates distributed between depths of about 20 m to 350 m. The same stratification scheme has been used since 1971, with the exception of the addition of three inshore strata (401 to 403) in 1984. The analyses are presented here for the 24 strata (415 to 439) sampled since 1971, representing an area of 70,091 km². The survey indices have been standardized for changes in survey vessels, gears, and protocols which have occurred over the time series using comparative fishing between vessels and gears. Stratified random survey indices are expected to be proportional to biomass and abundance for most species.
Data collection	This survey follows a stratified random sampling design, which covers an area of 73,214 km² and includes sampling of fish and invertebrates using a bottom trawl (Figure 1).<br>For the purpose of this study, we used 122 fish and crustacean taxa that were captured, identified and processed for total catch weight in the selected strata (Supplementary Table 1). Species were categorized based on their expected zonal vertical position within this marine ecosystem (demersal vs pelagic species), and if they are or were under a commercial fishery throughout the time series. Crustaceans included in the analysis consisted of several crab species, American lobster (Homarus americanus), and several decapod shrimp and prawn species (hereafter referred to as shrimps). Shrimps were analyzed as a group as species identification was inconsistent across the time series. Additionally the temporal range of shrimp data was limited to start in 1980 as these taxa were unreliably recorded prior to that year.
Timing and spatial scale	The analyses are presented here for the 24 strata (415 to 439) sampled since 1971, representing an area of 70,091 km². The analyses includes data collected from 1971 until 2021.
Data exclusions	The same stratification scheme has been used since 1971, with the exception of the addition of three inshore strata (401 to 403) in 1984. The analyses are presented here for the 24 strata (415 to 439) sampled since 1971, representing an area of 70,091 km².
Reproducibility	All analyses were conducted in the software environment s C2 and R version 4.2.2, and the package "rioja" was used for cluster analyses. The data used in this paper are available on the Government of Canada Open Data Portal. We provide computer code to facilitate reproducibility of the analysis. Reproducibility of data collection is done through consistent bottom trawling protocols. Since this study captures organisms in nature (rather than an experiment in the lab), the samples collected can never actually be reproduced.

Randomization	The study included marine organisms captured using a bottom trawl from a research vessel survey that follows a stratified random sampling design.
Blinding	Binding is not relevant to this study involving collection of marine organisms using a bottom trawl.

Did the study involve field work? ☒ Yes ☐ No

Field work, collection and transport

Field conditions	N/A, field work took place over the course of 51 years and were variable over the years.
Location	This study focuses on the sGSL, which is a shallow semi-enclosed sea that covers an area of approximately 85,000 km^2 south of the deep Laurentian Channel and is bordered by four Canadian provinces, Quebec, New Brunswick, Nova Scotia and Prince Edward Island (Figure 1).
Access & import/export	Sampling locations were accessed on a Canadian Coast Guard Research Vessel. All captured specimens were sorted by ID, counted and weighed. A small subsumable of some species were retained for biological samples and the rest were released back into the environment as soon as possible. All fishes were collected and retained under a License to Fish for Scientific Purposes, pursuant to Part VII, Section 52, of the Fisheries (General) Regulations of Canada. Fecundity
Disturbance	Bottom trawling disturbs the benthic marine habitat. Standard research bottom trawl protocols were followed to minimize harm to the seafloor habitat.

Reporting for specific materials, systems and methods

We require information from authors about some types of materials, experimental systems and methods used in many studies. Here, indicate whether each material, system or method listed is relevant to your study. If you are not sure if a list item applies to your research, read the appropriate section before selecting a response.

Materials & experimental systems

n/a	Involved in the study
☒	☐ Antibodies
☒	☐ Eukaryotic cell lines
☒	☐ Palaeontology and archaeology
☒	☐ Animals and other organisms
☒	☐ Clinical data
☒	☐ Dual use research of concern
☒	☐ Plants

Methods

n/a	Involved in the study
☒	☐ ChIP-seq
☒	☐ Flow cytometry
☒	☐ MRI-based neuroimaging

Plants

Seed stocks	NA
Novel plant genotypes	NA
Authentication	NA
